# Implementation of a Potential Field-Based Decision-Making Algorithm on Autonomous Vehicles for Driving in Complex Environments

**DOI:** 10.3390/s19153318

**Published:** 2019-07-28

**Authors:** Carlos Martínez, Felipe Jiménez

**Affiliations:** Instituto Universitario de Investigación del Automóvil (INSIA), Universidad Politécnica de Madrid, 28031 Madrid, Spain

**Keywords:** intelligent transport systems, autonomous vehicles, path-planning, vehicle perception

## Abstract

Autonomous driving is undergoing huge developments nowadays. It is expected that its implementation will bring many benefits. Autonomous cars must deal with tasks at different levels. Although some of them are currently solved, and perception systems provide quite an accurate and complete description of the environment, high-level decisions are hard to obtain in challenging scenarios. Moreover, they must comply with safety, reliability and predictability requirements, road user acceptance, and comfort specifications. This paper presents a path planning algorithm based on potential fields. Potential models are adjusted so that their behavior is appropriate to the environment and the dynamics of the vehicle and they can face almost any unexpected scenarios. The response of the system considers the road characteristics (e.g., maximum speed, lane line curvature, etc.) and the presence of obstacles and other users. The algorithm has been tested on an automated vehicle equipped with a GPS receiver, an inertial measurement unit and a computer vision system in real environments with satisfactory results.

## 1. Introduction

The benefits of autonomous driving can be huge [1]. For example, the number of traffic accidents would be reduced, since it is estimated that around 90% of accidents are caused by human errors [2]. The most widely accepted classification of autonomous vehicles is presented by SAE (Society of Automotive Engineers) [3]. It was published for the first time in January 2014, and the last published revision is dated June 2018. It defines six levels of automation (from 0 to 5), depending on the degree of attention and human intervention required for the driving tasks. Table 1 shows a summary.

There are several applications of autonomous vehicles in different environments. Even private vehicles are the most widespread among the public, autonomous vehicles are not limited to them [4,5]. One of the most remarkable examples in other sectors is the large-scale goods transport. In order to increase road capacity and companies’ productivity, a method has emerged consisting of the grouping of a set of vehicles as a platoon [6,7]. In addition to goods transport, the irruption of autonomous driving technology provides solutions for public transport, such as autonomous-taxi models [8,9] and car-sharing [10,11]. Another sector in which autonomous driving is of great interest is the one of exploration, surveillance and military activities [12]. In this way, interventions could be carried out without risking human lives. Automated vehicles represent a technology that will create enhanced mobility opportunities for the elderly population and disabled people [13].

In summary, nowadays, the operation of autonomous vehicles is a reality in simple environments, but presents some decision-making challenges in complex scenarios [4]. Some examples are environments with heavy traffic where it is necessary to carry out a lane-change, entering a highway, or entering a roundabout. These tasks are quite trivial for experienced human drivers, but are high difficulty for a machine that follows the driving rules strictly; does not use, in general, previous expectations to solve conflicts; and does not extract the same information from each scenario (making impossible a subjective assessment of that scenario).

In this paper, a potential-field model for decision making in changeable traffic scenarios has been adapted, adjusting its parameters and operation so that it can operate in an autonomous vehicle in real time. Furthermore, some modifications to the conventional definition of this kind of model have been introduced in order to solve some of the main limitations, such as the limited application of responses to future events. Driving tests have been carried out in real-life scenarios and realistic environments. The environment was unknown, and we had no control over any variable, which allows us to generalize the response of the system. The tests were performed using an autonomous vehicle instrumented and equipped with sensor systems.

The paper is organized as follows: Section 2 discusses related works on autonomous cars and path planning techniques. Section 3 explains all the theoretical aspects of the potential-field model. Section 4 provides a description of the hardware and software architectures of the vehicle in which the decision algorithms are implemented for tests on real roads. Section 5 presents the different tests carried out, as well as the results obtained. Finally, Section 6 presents the main conclusions of this paper.

## 2. Related Work

Developing an autonomous vehicle involves three main elements: Perception (in a general way, including positioning and communications if available), decision making and action. The last one is perhaps the simplest one, so little research is required now, and most developments are now focused on the first and second ones. Concerning perception, the technology has undergone a great deal of development in recent years. Autonomous vehicles could use a wide variety of sensor systems, such as computer vision [14,15], ultrasonic [16], radar [17] or LiDAR [18,19], among others. Thanks to the advantages and limitations of each one, the current trend is focused on sensor information fusion [20,21]. Furthermore, vehicular communications enhance the electronic horizon, and satellite positioning on a digital map provides information as a secondary sensor.

The next step is decision-making. From a classical point of view, driving must address decisions at three different levels [22]. The first one involves the tasks related to navigation, such as choosing an appropriate route to reach the selected destination. The second one consists of strategies while driving (adjustment of the speed according to the environment, safe distance keeping from the other vehicles, or criteria to perform the lane-change maneuver). Finally, the third one refers to the operation of the actuators: Pedals (accelerator and brake), gear shift and steering wheel.

Nowadays, vehicles with some level of automation can perform simple and repetitive control tasks such as lane tracking, or speed/safety distance maintenance, but they are not prepared to deal with complex and changing scenarios or with uncertainties. Therefore there is still the technological challenge of having more generic decision strategies, but, at the same time, running in real time and maintaining very high reliability standards.

Therefore, decision-making involves a multitude of aspects [4,23,24] (route planning, the road type and geometry, the presence of different elements on the road-obstacles, pedestrians, other vehicles—and comfort criteria, among others).

In addition, the scenarios that must be faced can be very varied. This implies that techniques and processes that can be systematically applied to decision-making must be as general as possible in order to face unexpected changes of the environment. Furthermore, the amount of data to be processed is very high and responses must be developed with very strict time requirements. Autonomous driving requires work with real-time applications. In summary, decision making needs to meet strict performance, safety, and fault tolerance criteria. So, there are different types of path-planning techniques which can be used in the navigation of autonomous vehicles. Perhaps, one of the simplest approaches is to use a target assignment strategy to allow the vehicle to navigate through successive waypoints in the environment [25].

Evolutionary algorithms have demonstrated to be very useful in tasks related to vehicle control and route calculation. A strategy for identification and correction of odometry data using evolutionary algorithms is presented in [26]. Within the evolutionary algorithms, there are two techniques for path planning in robotic applications which stand out: Genetic algorithms and particle swarm optimization. A comparison of parallel genetic algorithms and particle swarm optimization algorithms is made for path planning in real time robotic applications in [27].

In addition, there have been many recent developments in optimization-based planning methods. Besides evolutionary algorithms, there have been many recent planning approaches based on nonlinear optimization [28] and model predictive control [29].

Path planning strategies based on fuzzy logic have demonstrated to be a very versatile tool and widely used in different parts of vehicle control. For example, an overtaking system for autonomous vehicles that uses fuzzy controllers that mimic human behavior and reactions during overtaking maneuvers is presented in [30]. A system for pedestrian collision avoidance (at speeds up to 30 km/h) in autonomous vehicles using a fuzzy steering controller is presented in [31].

Nowadays, the application of techniques such as deep learning or neural networks in applications of this type are undergoing great developments as well. For example, a spiking neuron path planning algorithm for an autonomous robot that can adjust its routes depending on the environment is introduced in [32].

Rapidly-exploring random tree (RRT) algorithms are used in the calculation of path planning in robotic applications, as well as in vehicles [33,34]. Besides that, algorithms based on A* technique have been widely used in pathfinding problems [35,36].

Reactive techniques compute their response at each instant based on the current environment and can face highly dynamic and unpredictable environments [37]. Within the reactive techniques, planning based on artificial potential fields can be highlighted. It is a quite common technique in robotics, but its use in autonomous driving is not yet so widespread [38].

Some of the theoretical limitations of these techniques in robotics are presented in [39]. One of the main limitations of systems based on algorithms with potential fields would be the possible presence of local minima, but there are different approaches to avoid this problem, like the concept of the virtual obstacle for local minima recovery in [40,41] or the harmonic potential functions in [42].

Finally, the use of communications between vehicles (V2V) or between vehicles and infrastructure (V2I) is currently undergoing a great development [43]. Using communications, more information is available for path calculation and adapting speed [44].

The main contribution of this paper is the implementation of a generic path planning model, based on potential fields, on autonomous vehicle so it could react to unstructured situations. It is proposed to use a generic driving technique that can deal with any type of event, rather than determinist methods such as those currently applied. This model is specifically designed for road vehicles and not for robotic applications, and this fact has clear implications on the model definition and parameter fitting process. Furthermore, compared to conventional algorithms, some modifications are introduced in order to overcome the limitations regarding future events in the vehicle path. Tests have been carried out in real scenarios (apart from previous simulated data), specifically on highways with an instrumented autonomous vehicle. In the tests carried out, the estimation of the speed and steering angle were checked. The scenarios in which the tests were performed were not known beforehand, nor did we have control over any of their variables. The fact of not having control over the scenario allowed us to prove the generalizability of the proposed system. 

## 3. Decision Making Algorithm

Path-planning based on potential models is a tool that is included within the reactive techniques. They are based on the fact that an object (in this case the vehicle) reacts at all times to the current situation and environment, generating an appropriate movement proportional to that situation. The selection of this kind of technique has the clear drawback that, in general, only the present information around the vehicle is considered, appropriate responses being generated from it. But other advantages make it a good solution. It is quite computationally efficient, and decisions can be obtained in real time with very high sampling rates. Furthermore, decisions are directly linked to physical variables so they are easily comprehensible, and this fact is essential for avoiding strange behaviors of the autonomous vehicle that could surprise other road users.

**Figure 1:**
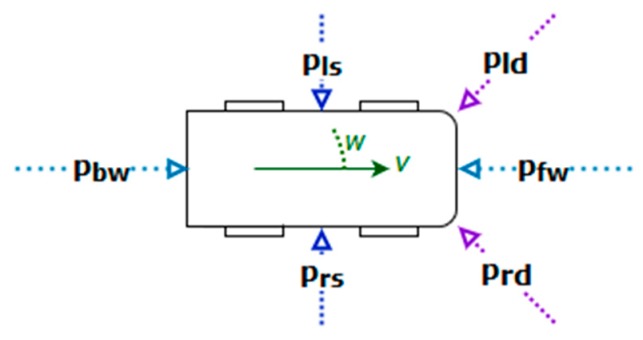
General schema of the different potential field sources.

In the path-planning technique based on potential models, each element of the environment (lanes, other vehicles, obstacles, etc.) produces a potential field (attraction or repulsion) in an analogue way to what magnetic charges do (depending on whether their mathematical sign is positive or negative). That is, the vehicle will react to the presence of the different potential fields (Figure 1) with an appropriate and proportional behavior. Depending on the different potentials considered each time, the vehicle modifies its movement by acting on the steering wheel, the throttle or the brake pedals.

In the following sections, the different possible types of potential sources will be explained: Forward, backward, side and diagonal potentials.

### 3.1. Forward and Backward Potentials

**Figure 2:**
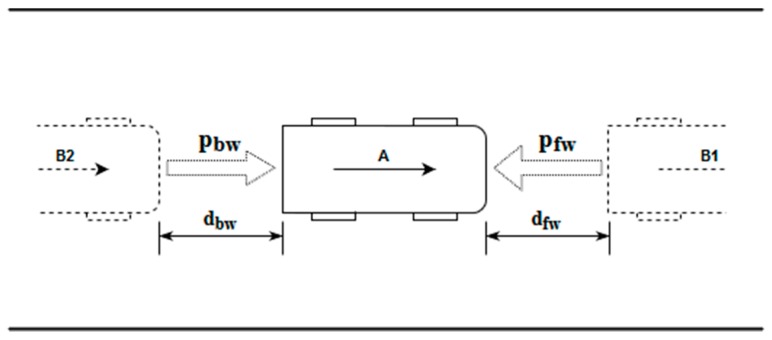
Scheme for the forward and backward potential fields calculation.

The first potential sources that must be considered are forward and backward potentials. Figure 2 shows a scheme of both situations, as well as the variables involved in their calculation. In this scheme, there are two vehicles (vehicle B1 and vehicle B2) interacting with the ego vehicle (vehicle A).

On the one hand, the forward potential field behaves in such a way that other obstacles that are directly in front of the vehicle will produce changes in it. That is, in Figure 2, vehicle B1 (which drives in front of vehicle A) generates a potential field that affects the behavior of the ego vehicle. The forward potential (p_fw_) is calculated by Equation (1):

(1) $$p_{\mathrm{fw}}=\{\begin{array}{ll} 0, & {\mathrm{for}\;v}_{B1}\geq v_{\max}\\ {(v}_{\max}{-v}_{B1}{)/d}_{\mathrm{fw}}, & {\mathrm{for}\;v}_{B1}{<v}_{\max} \end{array}$$

where $v_{B1}$ is the speed of the obstacle, $v_{\max}$ is the maximum road speed and $d_{\mathrm{fw}}$ is the distance measured on the longitudinal axis of the road between the vehicle and the obstacle.

On the other hand, the backward potential (p_bw_) is the one that is produced due to the presence of vehicles or other obstacles in the vicinity of the rear (e.g., vehicle B2 in Figure 2), as described in Equation (2):

(2) $$p_{\mathrm{bw}}=\{\begin{array}{ll} 0, & {\;\mathrm{for}\;v}_{A}\geq v_{B2}\\ {(v}_{B2}{-v}_{A}{)/d}_{\mathrm{bw}}, & {\;\mathrm{for}\;v}_{A}{<v}_{B2} \end{array}$$

This potential is used as an element to foster cooperation between vehicles, so that if the vehicle behind it (at a distance $d_{\mathrm{bw}}$) has a higher speed ($v_{B2}$) than the autonomous vehicle ($v_{A}$), it will help vehicle B2 to perform the overtaking maneuver, whenever possible.

### 3.2. Side and Diagonal Potentials

**Figure 3:**
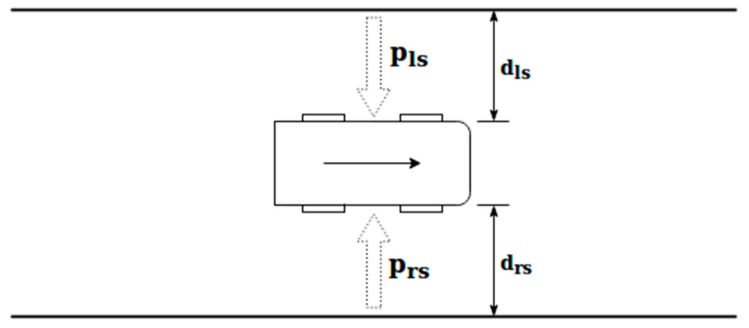
Scheme for the side potential field calculation.

The side potential is produced due to the presence of other elements on the sides of the vehicle (such as other vehicles or obstacles), or due to the road or lane lines. Figure 3 shows an example of the calculation of the variables that affect the side potential, without the presence of obstacles, that is, taking into consideration only the lane lines.

The value of the side potential (p_s_) can be calculated at every moment by Equation (3):

(3) $$p_{s}=p_{\mathrm{rs}}+p_{\mathrm{ls}}=\frac{1}{d_{\mathrm{rs}}^{2}}-\frac{1}{d_{\mathrm{ls}}^{2}}$$

where $d_{\mathrm{ls}}$ and $d_{\mathrm{rs}}$ are the distances between the vehicle and the different elements located on its left and right sides, respectively, measured on the transverse axis of the lane. 

**Figure 4:**
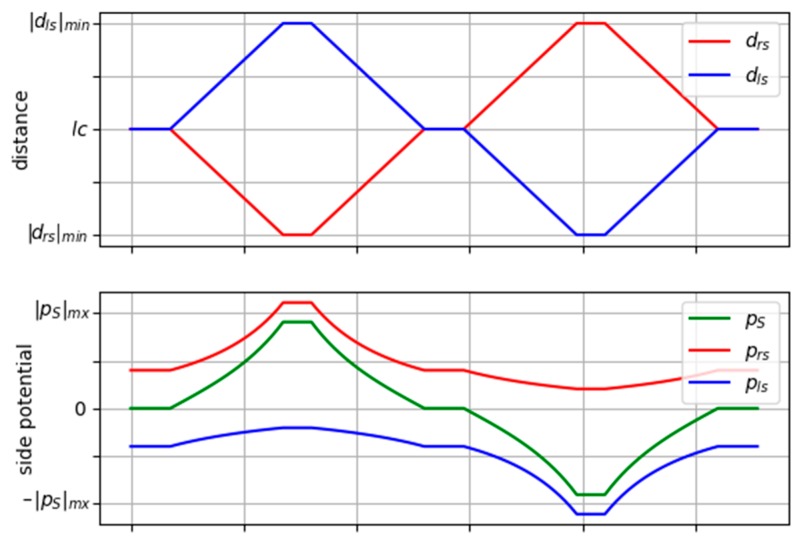
Example of the variation of the side potential field.

Figure 4 shows how the lateral potential field varies according to the distance to each of the limits of the lane. It is shown in red, the effects of the right side and in blue the effects of the left one (both distance and partial potential). In the figure, it can be verified that, when the vehicle drives in the center of the lane (lc), the potential takes a null value. However, if it approaches the left limit of the lane (firstly) or the right limit (secondly), the lateral potential takes the maximum values.

**Figure 5:**
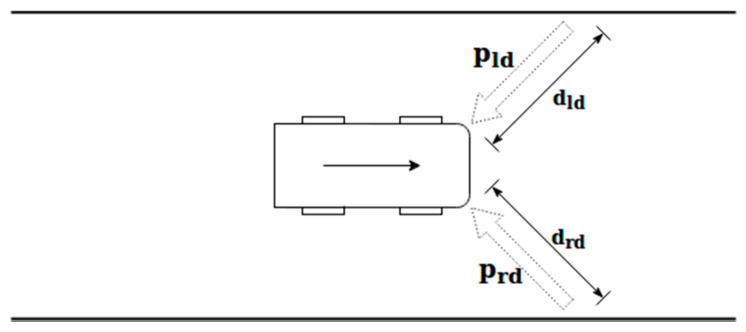
Scheme for the diagonal potential field calculation.

The diagonal potential is used to predict the vehicle position in future instances and anticipate future changes of the environment, so it can be used to make corrections in advance. Figure 5 shows an example of this type of potential field and also all the variables involved in its calculation.

The value of the diagonal potential field (p_d_) can be calculated by Equation (4) as a function of the front-left diagonal ($d_{\mathrm{ld}}$) and the front-right diagonal ($d_{\mathrm{rd}}$):

(4) $$p_{d}{=p}_{\mathrm{rd}}+p_{\mathrm{ld}}=\frac{1}{d_{\mathrm{rd}}^{2}}-\frac{1}{d_{\mathrm{ld}}^{2}}$$

It is necessary to consider the distance from each one of the front corners of the vehicle to the different obstacles or elements located in front of the vehicle (assuming a direction of ± 45 degrees with respect to the longitudinal axis of the vehicle).

### 3.3. Elements That Generate Potentials

The definition of those elements that are relevant each time for the vehicle decision making process and that are used for the definition of the aforementioned potential fields is a key issue. The most classical and simple approaches consider only obstacles and road or lane lines. A more complex approach considers traffic signals, such as speed limits of stop signals. However, this scope limits the ability of the algorithm from considering some other information that could be relevant in subsequent instances. This is the case, for example, for the information provided by the strategic level [5]; e.g., the presence of exit or merging ramps near the vehicle position, or the decision of taking a specific road exit. For this reason, the algorithm considers all the information available at each moment (not only the information provided by onboard sensors and the current scenario), useless data are automatically discarded because of their limited impact of the generated field. This strategy solves the main limitation of reactive decision-making methods and enhances the knowledge of the digital sight distance to make a decision.

### 3.4. Lateral and Longitudinal Control

Finally, the steering angle (θ) is calculated by applying a conversion factor (K_T_) to the calculated total potential (p_T_), according to Equation (5). This is what is known as lateral planning.

(5) $${\theta =K}_{T}\cdot p_{T}$$

where the total potential is the pondered sum of all the individual potentials, according to Equation (6):

(6) $$p_{T}=\sum K_{i}\cdot p_{i}{=K}_{s}\cdot p_{s}{+K}_{d}\cdot p_{d}{+K}_{\mathrm{fw}}\cdot p_{\mathrm{fw}}{+K}_{\mathrm{bw}}\cdot p_{\mathrm{bw}}$$

where:

- K_s_ governs the lateral sensitivity of the vehicle. A high value means that small potential fields will make large changes in the steering angle.
- K_d_ is used to control the effect of the next lateral corrections.
- K_fw_ governs the longitudinal sensitivity of the vehicle. High values will cause the vehicle to avoid the obstacles very soon.
- K_bw_ controls the way the vehicle cooperates with others.

On the other hand, the speed of the vehicle is calculated in such a way that it drives at the maximum possible speed considering the movement of other road users, making sure to do it safely, respecting a comfortable deceleration (a) and without exceeding the maximum speed allowed on the road ($v_{\max}$). This is what is known as longitudinal planning (Equation (7)).

(7) $$v=\min(\sqrt{v_{B1}^{2}+2\cdot a\cdot d_{\mathrm{fw}}}{,\;v}_{\max})$$

It can be checked how, apart from respecting the speed limits, the speed v must be adequate with other vehicles in the lane (v_B1_), so that there is no risk of collisions.

## 4. Experimental Testbed Platform

The decision-making algorithm was tested in simulated and real scenarios. For the latter, an autonomous vehicle with open hardware and software architectures was used, in which new decision algorithms could be implemented. Those architectures are described in this section.

### 4.1. Hardware Architecture

**Figure 6:**
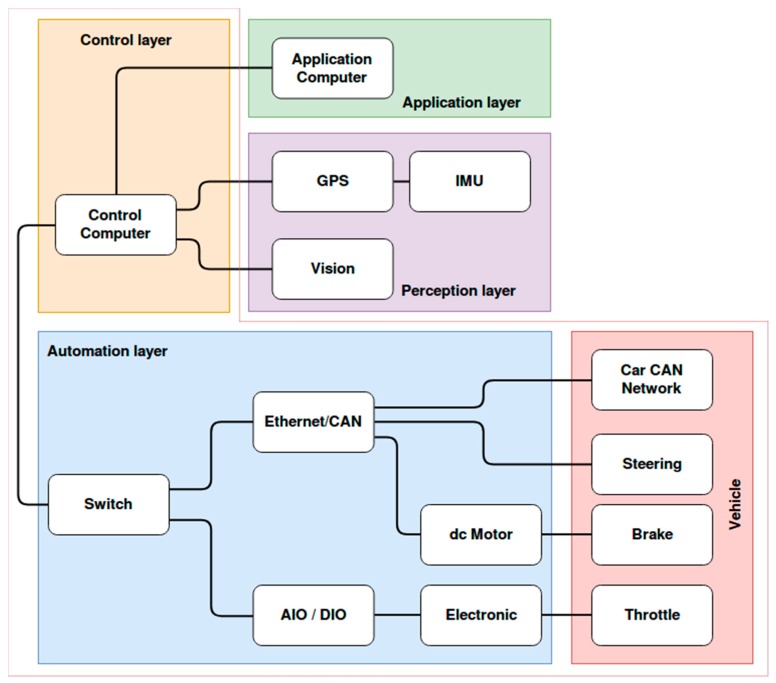
Diagram of the hardware architecture layers.

The hardware architecture of the proposed system consists of five layers: Vehicle, automation, control, perception and application, as shown in Figure 6. The vehicle layer represents the car on which the different sensors are embedded and whose behavior is controlled with the decision model. It contains, in a very summarized way, a CAN (Controller Area Network) of the vehicle and the three actuators. The automation layer is responsible for interacting with the vehicle’s actuators (accelerator, brake and steering wheel) so that these can be controlled by a computer. This layer includes all the necessary electronics to act on the actuators. The control layer includes the computer that manages the low-level control of the elements of the automation layer. That is, it receives mainly two input variables—speed and steering angle. Using these inputs, it is responsible for generating the appropriate signals and messages for the automation layer to work correctly and safely. This layer serves as a communication link between the rest of the layers and with the automation layer (and therefore, with the car). The perception layer incorporates all the sensor systems: GPS (Global Positioning System) receiver, an IMU (Inertial Measurement Unit) and a computer vision system. Finally, the Application layer includes high-level tasks (path-planning, behavior management, data calculation and visualization, etc.).

### 4.2. Software Architecture

**Figure 7:**
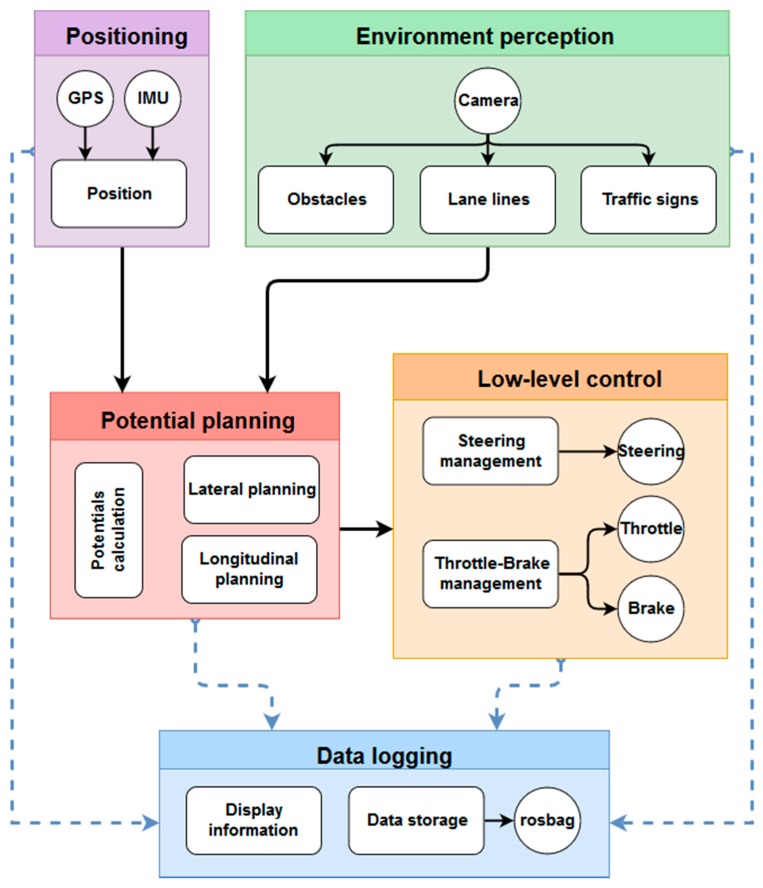
General diagram of the software architecture layers.

This section shows the different nodes that constitute the software architecture in which any decision-making algorithm could be implemented. The system is composed of five nodes—positioning, environment perception, potential planning, low-level control and data logging, as shown in Figure 7. It shows that the Positioning and Environment perception nodes obtain information that is sent to the potential planning node, which is connected to the low-level control node. There is also a node (data logging), which is responsible for collecting data from the rest of the nodes (blue dashed line) and stores or displays them, as appropriate. 

Firstly, the positioning node receives data from the GPS system and the inertial measurement unit (IMU). With all these data, its main task is carrying out accurate vehicle positioning. Secondly, the environment perception node is responsible for processing the computer vision system. Using the images that it receives, it provides information about the road lines (position, curvature, etc.), about possible obstacles on the road (pedestrians or other vehicles) and about the different traffic signals (mainly, speed limit signs). Then, the potential planning node is responsible for calculating the potential fields. With all these data, the tasks of lateral planning (calculation of the steering angle) and longitudinal planning (calculation of speed) are carried out. Afterwards, the node low-level control receives the values of steering angle target and speed target. With this information, the node manages the steering wheel, throttle and brake, in order to achieve the target values. Finally, the data logging layer is responsible for storing the calculated data and showing the information to the user. 

## 5. Tests

This work has adapted a path planning model based on potential fields so that it can work in real environments. Its behavior has been adapted and adjusted to be used on real roads. Working in real environments requires the ability of facing unexpected changes in the variables of the environment (for example, vehicles that do not drive in a typical way or elements that make it difficult to detect the road lines). In addition, the amount of data to be considered and to be processed is much bigger, so processing and response time requirements are quite strict. Therefore, the system must be robust and fast enough to achieve a proper performance.

### 5.1. Simulated Scenarios Definition

**Figure 8:**
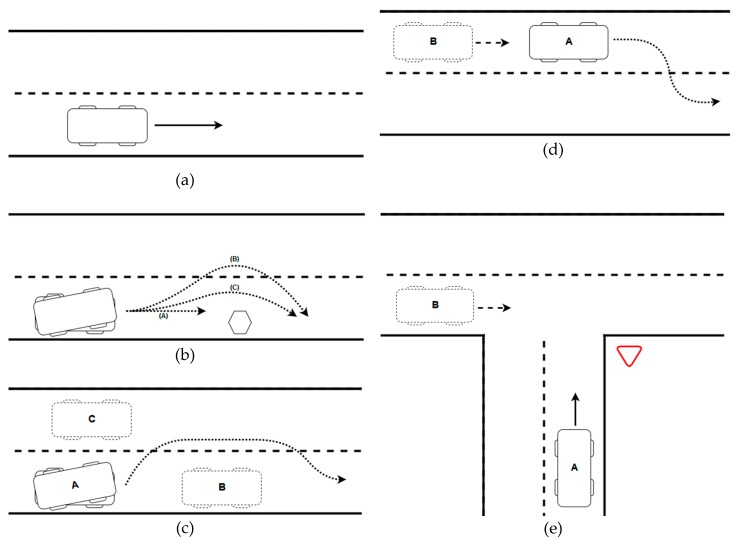
Simulated scenarios. (**a**) Lane maintenance; (**b**) obstacle in the lane; (**c**) overtaking maneuver; (**d**) overtaking facilitating; (**e**) crossing approach.

The parameters involved in the calculation of the potential fields should be adjusted in order to obtain realistic and feasible responses of the vehicle. For this task, different tests were carried out in simulated scenarios. Furthermore, correct behavior of the model should be assessed previously to the real tests with an autonomous vehicle. All these scenarios are shown in Figure 8.

### 5.2. Parameters Analysis

The effect of modifying each parameter should be characterized in simulations, so they are varied in such a way as to obtain adequate responses, and to not produce abrupt or unstable behaviors.

The process of adjusting the configuration parameters is carried out as follows. Firstly, the K_s_ parameter is adjusted in an environment without obstacles so that the vehicle drives properly, meaning that it is correctly centered in the lane. In addition, these changes had to be proportional, because very high values of K_s_ would produce very abrupt responses and could make the vehicle unstable.

The K_d_ parameter is then gradually increased so that the vehicle can anticipate the effect of near future lateral variations. The value of this parameter cannot be very high, since its objective is to anticipate near future actions, but other potential fields are responsible for performing the maneuver.

Once the K_s_ and K_d_ parameters have been adjusted, the vehicle will be able to drive properly in the lane and face some situations that may occur. The next step is to set the value of the K_f_ parameter so that the vehicle can react to obstacles in front of it. Its value must allow for facing the obstacles, but without producing abrupt outputs: High values will cause the vehicle to avoid the obstacles very soon.

Due to one of the behavioral rules of the autonomous vehicle being to drive along the right lane (slow lane) whenever possible, after an overtaking maneuver, it returns to the original lane as soon as this is safe enough. For this reason, it is not necessary to adjust the K_b_ parameter.

Finally, Table 2 shows a summary of the effect of modifying the configuration parameters. In general, increasing these values will produce very abrupt responses and instability in the system. On the other hand, decreasing these values will impede the system from achieving an adequate response.

### 5.3. Tests in Real Environments

**Figure 9:**
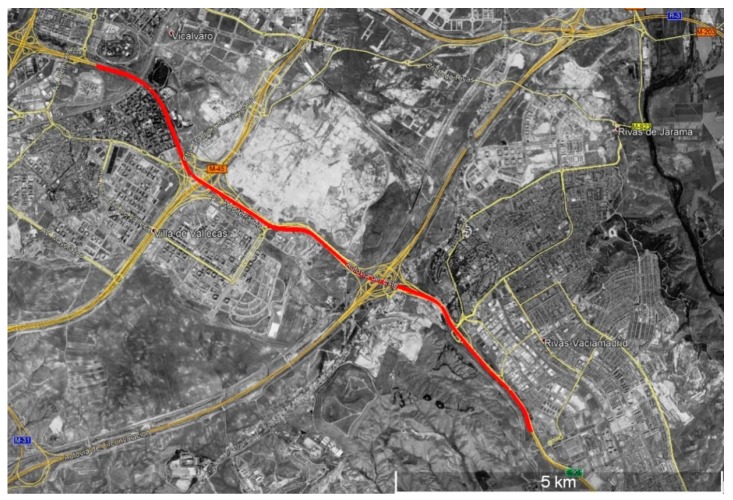
Highway test area.

In order to assess the system in a practical and real way, different tests were performed to evaluate the strengths, weaknesses and possible improvements. The tests were carried out on the E-901/A-3 highway in Madrid, specifically between exits 7 and 17. Figure 9 shows a map of the test area.

The results obtained for the tests carried out are shown below. Firstly, an example of the calculation of the steering angle from the potential fields is presented. Secondly, the calculation of the speed from the potential field generated by a vehicle driving in front is shown. Data and decisions are made at a sampling rate of 5 Hz.

**Figure 10:**
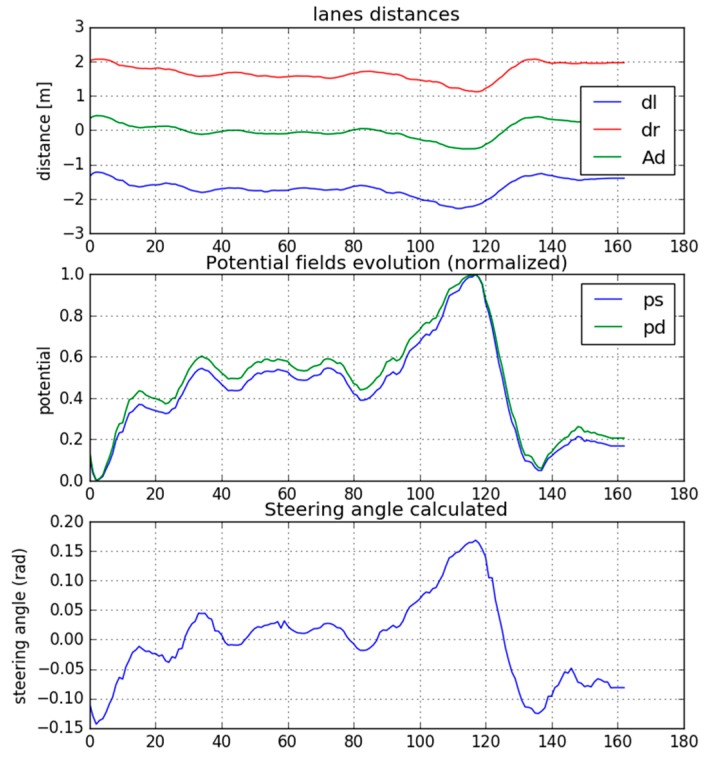
Lateral planning using potential fields.

Figure 10 shows the results obtained for the stretch of road the tests were performed on. The calculation of the steering wheel angle, as a function of lateral and diagonal potential, is shown graphically. The distance to each lane (d_l_ = left, d_r_ = right) is shown, as well as the calculation of the position of the center of the lane (A_d_) and the variation of the active normalized potential fields (lateral and diagonal). 

In this case, there were not obstacles, so only the potential fields generated by the lane lines are considered. This way, it can be checked how the lateral potential provides proportional values for driving in the center of the lane.

The system produces a response so that the steering wheel faces deviations when driving in the lane. In this way, the steering angle is calculated to drive centered in the lane. Generally, for small deviations, the system calculates turning angles of approximately ±0.05 rad. In this case, for slightly larger deviations, the calculated steering angles reach ±0.15 rad, a coherent value in this kind of environment.

It should be noted that the perception systems, such as the computer vision system, can provide false or inaccurate information. Then, potentials are not correctly calculated and abrupt changes in the target steering angle could appear. This is the reason why sensor fusion (GPS, IMU and LiDAR) is advisable to avoid these situations. 

**Figure 11:**
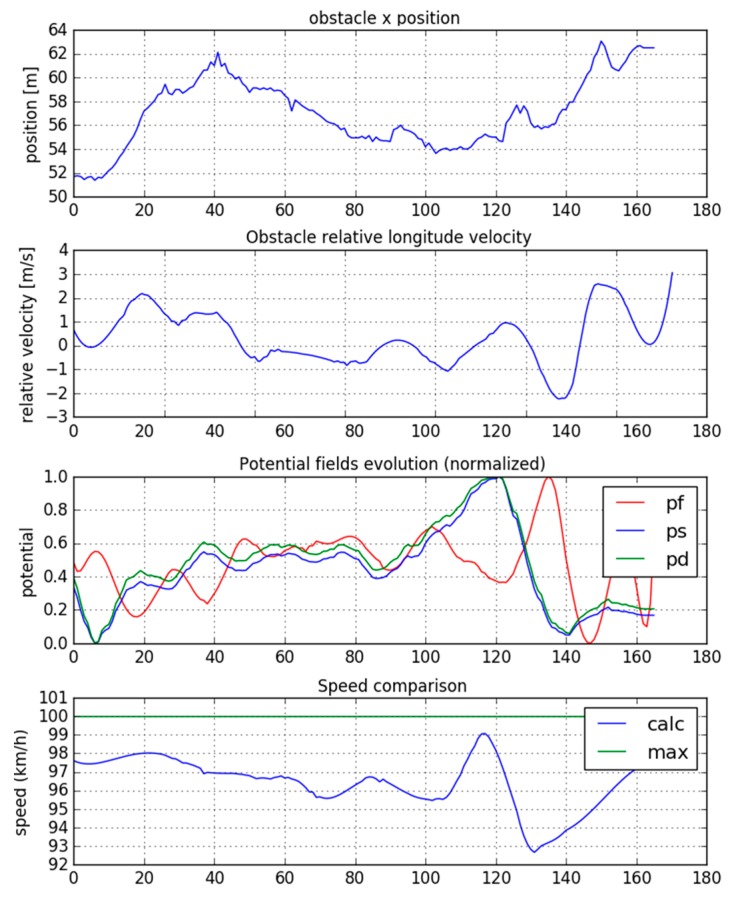
Longitudinal planning using potential fields.

Finally, Figure 11 shows the results obtained for the longitudinal planning calculation. In this case, there is another vehicle driving in front of the test car. This vehicle generates a potential field that affects the speed calculation. 

The distance at which the vehicle is located, the relative speed and the calculated potential fields (p_f_ = forward, p_s_ = side, p_d_ = diagonal) are shown. Using this information, target speed is calculated, below the maximum speed limit, considering the appropriate distance between the vehicle and the obstacle and the relative speed. In this case, the calculated speeds range between 90–100 km/h.

It is therefore demonstrated that the decision-making algorithm can operate on a real road, (specifically on a motorway) with real traffic flow, and that it would adapt correctly to changing scenarios. It should be kept in mind that tests were carried out in real and uncontrolled environments in which none of the scenario variables were previously known or predefined, so the vehicle could have found any possible situation and must react to them correctly.

## 6. Conclusions

In this paper, an algorithm for path-planning, based on potential fields, valid to react to any possible situation has been implemented, improving performance in comparison to current applications on autonomous vehicles. These potential fields are calculated from sensor data and obtained in real time. The tests to verify operation in real environments have been performed using an instrumented vehicle equipped with GPS, IMU and a computer vision system. These tests have been carried out in different stretches of a highway opened to traffic. The results were obtained from an unknown environment in which we had no control over any variable. This allowed us to prove the ability of generalizing the algorithm response because the scenario was not controlled.

For possible improvements, it is proposed to make use of LiDAR to improve the robustness and reliability in the perception of the environment, mainly because of false or limited information that computer vision can provide under highly demanding scenarios.

Now, more challenging scenarios, such as overtaking maneuvers and entering roundabouts are being tested on real roads. It should be noted that, in this case, after the algorithms decide to make a movement, vehicle dynamics models are used to obtain speed and steering angel target values, achievable in a physically safe way.

Finally, as a contribution to reactive models, variables of the next road stretches are also considered in potential calculations, so road exits, desired route, traffic expectations, etc., could modify decisions. In that sense, the use of V2X technologies in the following tests is proposed. The use of cooperative driving would allow vehicles to inform others where they are, as well as share information on the different obstacles they detect. In this way, the information would be more complete; therefore, maneuvers could be carried out more safely and efficiently. That fact overcomes the limitations of these kinds of models that only consider the local state of the scenario each time.

## Figures and Tables

**Table 1:** Levels of automated driving for road vehicles defined by the Society of Automotive Engineers (SAE).

SAE Level	Name	Control of Actuators	Monitoring of Environment	System Capability
0	No automation	Human driver	Human driver	n/a
1	Driver Assistance	Both	Human driver	Some DM ^1^
2	Partial Automation	System	Human driver	Some DM ^1^
3	Conditional Automation	System	System	Some DM ^1^
4	High Automation	System	System	Some DM ^1^
5	Full Automation	System	System	All DM ^1^

^1^ DM: Driving Modes.

**Table 2:** Analysis of the effect of the configuration parameters.

Parameter	Low Values	High Values
K_s_	Smooth response to changes in the environment	Abrupt response to changes in the environment
K_d_	Little anticipation to changes in the environment	High impact of future steps on the calculation of the current response
K_fw_	Late positioning to avoid an obstacle	Early positioning to avoid an obstacle

## References

[1] European Commission (2016). Gear 2030 Discussion Paper—Roadmap on Highly Automated Vehicles.

[2] National Highway Traffic Safety Administration (2015). Critical Reasons for Crashes Investigated in the National Motor Vehicle Crash Causation Survey.

[3] SAE International (2018). J3016: Taxonomy and Definitions for Terms Related to Driving Automation Systems for On-Road Motor Vehicles.

[4] Ziegler J., Bender P., Schreiber M., Lategahn H., Strauss T., Stiller C., Dang T., Franke U., Appenrodt N., Keller C.G. (2014). Making bertha drive—an autonomous journey on a historic route. IEEE Intell. Transp. Syst. Mag..

[5] Jiménez F. (2018). Intelligent Vehicles: Enabling Technologies and Future Developments.

[6] Jia D., Lu K., Wang J., Zhang X., Shen X. (2016). A survey on platoon-based vehicular cyber-physical systems. IEEE Commun. Surv. Tutorials.

[7] Santini S., Salvi A., Valente A.S., Pescapé A., Segata M., Lo Cigno R. (2017). A consensus-based approach for platooning with intervehicular communications and its validation in realistic scenarios. IEEE Trans. Veh. Technol..

[8] Greenblatt J.B., Saxena S. (2015). Autonomous taxis could greatly reduce greenhouse-gas emissions of US light-duty vehicles. Nat. Clim. Change.

[9] Bischoff J., Maciejewski M. (2016). Simulation of city-wide replacement of private cars with autonomous taxis in berlin. Procedia Comput. Sci.

[10] Spieser K., Treleaven K.B., Zhang R., Frazzoli E., Morton D., Pavone M. (2014). Toward a systematic approach to the design and evaluation of automated mobility-on-demand systems: A case study in singapore. Road Vehicle Automation.

[11] Fagnant D., Kockelman K., Bansal P. (2015). Operations of shared autonomous vehicle fleet for austin, texas, market. J. Trans. Res. Board.

[12] Naranjo J.E., Jimenez F., Anguita M., Rivera J.L. (2019). Automation kit for dual-mode military unmanned ground vehicle for surveillance missions. IEEE Intell. Transp. Syst. Mag..

[13] Harper C.D., Hendrickson C.T., Mangones S., Samaras C. (2016). Estimating potential increases in travel with autonomous vehicles for the non-driving, elderly and people with travel-restrictive medical conditions. Trans. Res. Part C.

[14] Bertozzi M., Broggi A., Fascioli A. (2000). Vision-based intelligent vehicles: State of the art and perspectives. Rob. Autom. Syst..

[15] Kastrinaki V., Zervakis M., Kalaitzakis K. (2003). A survey of video processing techniques for traffic applications. Image Vision Comput..

[16] Alonso L., Milanés V., Torre-Ferrero C., Godoy J., Oria J.P., de Pedro T. (2011). Ultrasonic sensors in urban traffic driving-aid systems. Sensors.

[17] Eltrass A., Khalil M. (2018). An Automotive Radar System for Multiple-Vehicle Detection and Tracking in Urban Environments. IET Intell. Transp. Syst..

[18] Kidono K., Miyasaka T., Watanabe A., Naito T., Miura J. Pedestrian recognition using high-definition LIDAR. Proceedings of the 2011 IEEE Intelligent Vehicles Symposium (IV).

[19] Jiménez F., Clavijo M., Castellanos F., Álvarez C. (2018). Accurate and detailed transversal road section characteristics extraction using laser scanner. Appl. Sci..

[20] Khaleghi B., Khamis A., Karray F.O., Razavi S.N. (2013). Multisensor data fusion: A review of the state-of-the-art. Inf. Fusion.

[21] Faouzi N.E., Klein L.A. (2016). Data fusion for ITS: Techniques and research needs. Trans. Res. Procedia.

[22] Díaz A., Clavijo M., Jiménez F., Talavera E., Serradilla F. (2018). Modelling the human lane-change acceptance behaviour through multilayer perceptrons and convolutional neural networks. Trans. Res. Part F.

[23] Li X., Sun Z., Cao D., He Z., Zhu Q. (2016). Real-time trajectory planning for autonomous urban driving: Framework, algorithms, and verifications. IEEE/ASME Trans. Mechatron..

[24] Glaser S., Vanholme B., Mammar S., Gruyer D., Nouveliere L. (2010). Maneuver-based trajectory planning for highly autonomous vehicles on real road with traffic and driver interaction. IEEE Trans. Intell. Transp. Syst..

[25] Vilca J., Adouane L., Mezouar Y. (2015). A novel safe and flexible control strategy based on target reaching for the navigation of urban vehicles. Rob. Autom. Syst..

[26] Mutz F., Veronese L.P., Oliveira-Santos T., de Aguiar E., Auat Cheein F.A., Souza F.D. (2016). Large-scale mapping in complex field scenarios using an autonomous car. Expert Syst. Appl..

[27] Roberge V., Tarbouchi M., Labonte G. (2013). Comparison of parallel genetic algorithm and particle swarm optimization for real-time UAV path planning. IEEE Trans. Ind. Inf..

[28] Schulman J., Duan Y., Ho J., Lee A., Awwal I., Bradlow H., Pan J., Patil S., Goldberg K., Abbeel P. (2014). Motion planning with sequential convex optimization and convex collision checking. Int. J. Rob. Res..

[29] Chang L., Seungho L., Varnhagen S., Tseng H.E. Path planning for autonomous vehicles using model predictive control. Proceedings of the 2017 IEEE Intelligent Vehicles Symposium (IV).

[30] Naranjo J.E., Gonzalez C., Garcia R., de Pedro T. (2008). Lane-change fuzzy control in autonomous vehicles for the overtaking maneuver. IEEE Trans. Intell. Transp. Syst..

[31] Fernandez Llorca D., Milanes V., Parra Alonso I., Gavilan M., Garcia Daza I., Perez J., Sotelo M.Á. (2011). Autonomous pedestrian collision avoidance using a fuzzy steering controller. IEEE Trans. Intell. Transp. Syst..

[32] Hwu T., Wang A.Y., Oros N., Krichmar J.L. (2018). Adaptive robot path planning using a spiking neuron algorithm with axonal delays. IEEE Trans. Cognitive Dev. Syst..

[33] Kuwata Y., Teo J., Fiore G., Karaman S., Frazzoli E., How J.P. (2009). Real-time motion planning with applications to autonomous urban driving. IEEE Trans. Control Syst. Technol..

[34] Zhang L., Manocha D. An efficient retraction-based RRT planner. Proceedings of the 2008 IEEE International Conference on Robotics and Automation 2008.

[35] Kala R., Shukla A., Tiwari R. (2010). Fusion of probabilistic A* algorithm and fuzzy inference system for robotic path planning. Artif. Intell. Rev..

[36] Stentz A. (1994). Optimal and efficient path planning for partially-known environments. Intelligent Unmanned Ground Vehicles.

[37] Kala R. (2016). On-Road Intelligent Vehicles.

[38] Padilla M.A., Savage J., Solís A., Aranbula F., Jing X.-J. (2008). Local Autonomous Robot Navigation Using Potential Fields. Motion Planning.

[39] Koren Y., Borenstein J. (1398–1404). Potential field methods and their inherent limitations for mobile robot navigation, In Proceedings of the 1991 IEEE International Conference on Robotics and Automation, Sacramento, CA, USA, USA, 9–11 April 1991; Volume 2, pp.

[40] Liu C., Ang M.H., Krishnan H., Lim S.Y. Virtual obstacle concept for local-minimum-recovery in potential-field based navigation. Proceedings of the 2000 ICRA. Millennium Conference. IEEE International Conference on Robotics and Automation. Symposia Proceedings (Cat. No.00CH37065).

[41] Zou X., Zhu J. (2003). Virtual local target method for avoiding local minimum in potential field based robot navigation. J. Zhejiang Univ. Sci. A.

[42] Kim J.-O., Khosla P.K. (1992). Real-time obstacle avoidance using harmonic potential functions. T-RA.

[43] Chen S., Hu J., Shi Y., Peng Y., Fang J., Zhao R., Zhao L. (2017). Vehicle-to-everything (v2x) services supported by LTE-based systems and 5G. IEEE Commun. Stand. Mag..

[44] Talavera E., Díaz A., Jiménez F., Naranjo J.E. (2018). Impact on congestion and fuel consumption of a cooperative adaptive cruise control system with lane-level position estimation. Energies.

